# Enhancing Object Detection in Underground Mines: UCM-Net and Self-Supervised Pre-Training

**DOI:** 10.3390/s25072103

**Published:** 2025-03-27

**Authors:** Faguo Zhou, Junchao Zou, Rong Xue, Miao Yu, Xin Wang, Wenhui Xue, Shuyu Yao

**Affiliations:** School of Artificial Intelligence, China University of Mining and Technology-Beijing, Beijing 100083, China; zhoufaguo@cumtb.edu.cn (F.Z.);

**Keywords:** object detection, coal mine, self-supervised pre-training, YOLO, feature extraction

## Abstract

Accurate real-time monitoring of underground conditions in coal mines is crucial for effective production management. However, limited computational resources and complex environmental conditions in mine shafts significantly impact the recognition and computational capabilities of detection models. This study utilizes a comprehensive dataset containing 117,887 images from five common underground mining tasks: mine personnel detection, large coal lump identification, conveyor chain monitoring, miner behavior recognition, and hydraulic support shield inspection. We propose the ESFENet backbone network, incorporating a Global Response Normalization (GRN) module to enhance feature capture stability while employing depthwise separable convolutions and HGRNBlock modules to reduce parameter volume and computational complexity. Building upon this foundation, we propose UCM-Net, a detection model based on the YOLO architecture. Furthermore, a self-supervised pre-training method is introduced to generate mine-specific pre-trained weights, providing the model with more semantic features. We propose utilizing the combined backbone and neck portions of the detection model as the encoder of an image-masking pre-training structure to strengthen feature acquisition and improve the performance of small models in self-supervised learning. Experimental results demonstrate that UCM-Net outperforms both baseline models and the state-of-the-art YOLOv12 model in terms of accuracy and parameter efficiency across the five mine datasets. The proposed architecture achieves 21.5% parameter reduction and 14.8% computational load decrease compared to baseline models while showing notable performance improvements of 1.3% (mAP_50:95_) and 0.8% (mAP_50_) in miner behavior recognition. The self-supervised pre-training framework effectively enhances training efficiency, enabling UCM-Net to attain an average mAP_50_ of 94.4% across all five datasets. The research outcomes can provide key technical support for coal mine safety monitoring and offer valuable technological insights for the public safety sector.

## 1. Introduction

Coal, as one of the most abundant fossil fuels worldwide, serves as a primary energy source for numerous countries and regions [1,2,3]. However, coal mining operations are frequently conducted under harsh and high-risk working conditions, where improper human operations may trigger severe mine accidents [4]. Simultaneously, equipment malfunctions and coal blockages during production processes significantly impact operational efficiency [5]. Therefore, rapid and precise detection of production processes and environmental conditions holds critical importance for ensuring mining safety and optimizing productivity.

The development of computer vision technology has led to the gradual adoption of machine-vision-based image recognition methods in underground coal mines, replacing traditional manual observation and monitoring approaches. These methods offer advantages such as low detection costs, high efficiency, strong real-time performance, and a high degree of automation, thus reducing the intensity of physical labor and minimizing subjective error rates [6]. Machine-vision-based mine image detection methods can be broadly classified into two categories based on feature extraction techniques: one consists of manual feature extraction methods using image processing and machine learning, and the other consists of automatic feature extraction methods based on deep learning. Among these, machine learning-based methods were the first to be developed and applied in the field of coal mine detection, achieving excellent results. For instance, He et al. [7] achieved accurate classification and monitoring of coal mine areas through the combination of a tree root algorithm and an extreme learning machine. Similarly, Dou et al. [8] constructed an efficient coal and gangue classification model using the Relief algorithm and support vector machines (SVMs), realizing automated and accurate coal and gangue sorting. However, machine learning methods rely heavily on handcrafted features, which are inefficient and have poor generalization ability.

In recent years, significant progress has been made in research on deep learning object detection in underground coal mine environments. In terms of innovation in algorithmic architecture, object detection algorithms have evolved from two-stage (such as SPPNet [9] and Faster R-CNN [10]) to single-stage algorithms (such as the YOLO series [11,12,13,14,15]), and researchers’ focus in the field of object recognition for mining applications has gradually shifted from two-stage algorithms to single-stage algorithms [16,17]. In terms of specific scene applications, some researchers have designed lightweight ore sorting networks LOSN [18], safety helmet wearing detection models DSM-YOLO [19], YOLO-BS models for conveyor blockage [20], and dedicated detection systems such as active-learning-based pedestrian detection models [21]. In terms of model improvement strategies, many researchers have introduced additional modules such as attention mechanisms, Transformers, and residual connections into detection networks. For example, Wang et al. [22] integrated deformable convolutions and applied offset learning to enhance fine-grained feature extraction. Wen et al. [23] introduced Swin Transformer blocks to build a lightweight coal and gangue detection network based on CNN and Transformer architectures. Xue et al. [24] used the ResNet18 network as the backbone feature extraction network and proposed the ResNet18-YOLO coal and gangue detection algorithm. It is not difficult to see that current underground coal mine detection research is still dominated by task-customized models, but existing methods still have significant shortcomings, specifically manifested in three aspects.

First, the limitations on application scenarios are prominent. Existing research mostly designs dedicated networks for single tasks such as sorting and safety helmet wearing detection, lacking a general detection framework that adapts to complex multi-task scenarios in mines. Second, the algorithm’s adaptability has shortcomings. Although mainstream single-stage algorithms improve detection efficiency through regression strategies, native YOLO and other models have insufficient feature extraction stability in low-illumination and masking scenarios in mines, resulting in poor detection accuracy. Third, model optimization strategies are unbalanced. Current model optimization often introduces additional modules or adopts complex architectures such as the GD mechanism [25] and GSConv-SlimNeck [26]. Although it improves multi-scale detection capabilities, the redundant design also greatly increases the model’s parameter count, failing to achieve a balance between accuracy and computational load in the limited-computing-power environment of mines.

With the success of unsupervised pre-training in NLP [27,28], self-supervised pre-training has also achieved significant progress in the field of image processing. He et al. [29] proposed MAE, which utilizes an asymmetric encoder–decoder structure to learn visual representations by masking image patches and reconstructing missing parts to achieve pre-training. Dai et al. [30] introduced UP-DETR, which is based on the DETR (Detection Transformer) model [31] and addresses multi-task learning and multi-query localization during pre-training. However, convolutional networks struggle with irregular and randomly masked inputs. To address this issue, SparK [32] and ConvNeXt-V2 [33] were used to explore masked-image modeling within convolutional architectures. ConvNeXt-V2, through collaborative masked autoencoding, significantly outperforms ConvNeXt [34] across multiple benchmarks. Meanwhile, SparK treats unmasked pixels as sparse voxels in a 3D point cloud and applies sparse convolution for hierarchical reconstruction, demonstrating strong feature transferability and greater benefits with larger networks. Despite the promise of generative pre-training in convolutional networks, self-supervised pre-training has yet to be applied to underground coal mine image tasks, and its effectiveness for lightweight models remains limited.

To address the above challenges, this paper proposes a universal underground coal mine object detection network, UCM-Net, which balances accuracy and real-time performance. Additionally, a self-supervised pre-training method is introduced to provide the model with richer feature information and further enhance detection accuracy. This study’s specific contributions to the literature are as follows:

- We propose a Hierarchical Global Response Normalization Block (HGRNBlock) to enhance the model’s feature expression capabilities and stability.
- We introduce depthwise separable convolution, combined with the Hierarchical Global Response Normalization module, to propose ESFENet, a feature extraction network for mine images. Based on the YOLOv8 model, we propose the underground coal mine object detection network, UCM-Net, which balances both precision and real-time performance for coal mine object detection.
- We design a self-supervised pre-training model structure for mine data based on the SparK masked encoder, generating dedicated pre-training weights for coal mine tasks for the first time.
- To address the limited benefits of self-supervised pre-training for lightweight models, we incorporate the feature fusion neck layer from the YOLO series into the pre-training structure. This enhancement enables the model to not only acquire more feature information during training but also achieve better fusion of these features.

## 2. Methods

In this section, we detail our approach to enhancing underground coal mine object detection by designing a network architecture that balances high accuracy with low resource consumption for real-time, multi-task detection while also employing self-supervised pre-training techniques to train model weights specifically for this environment, addressing the challenge that smaller models often gain limited benefits from traditional self-supervised pre-training methods by proposing a backbone-plus-neck structure for improved performance.

### 2.1. Lightweight Detection Network

#### 2.1.1. UCM-Net

**Figure 1:**
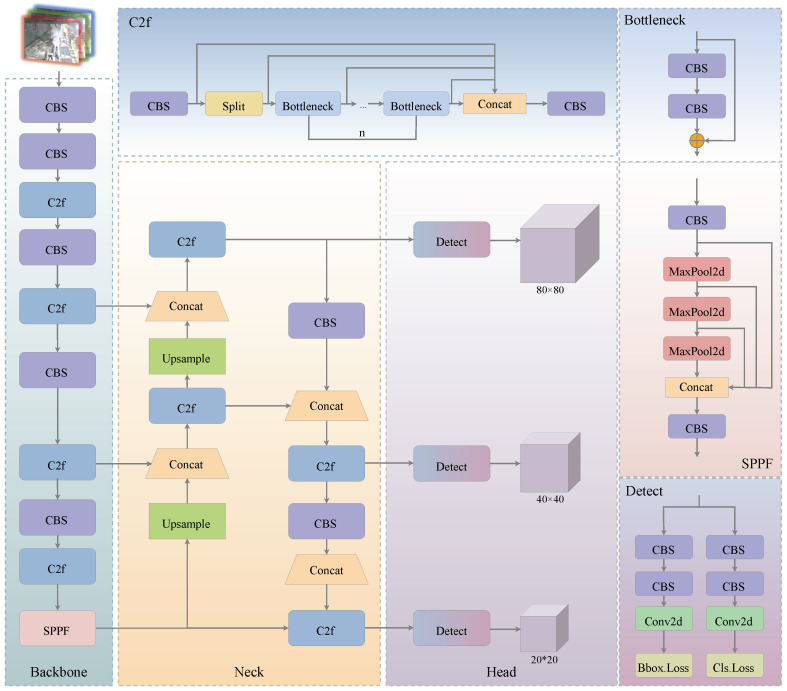
The structure of the proposed network, UCM-Net.

Considering the difficulty of feature extraction from underground images and the need for computational efficiency in detection tasks, we propose the Efficient and Stable Feature Extraction Network (ESFENet). Using ESFENet as the backbone and combining it with the YOLOv8n model, we build the Universal Underground Coal Mine Object Detection Network (UCM-Net). This architecture achieves model lightweighting and addresses the problem of insufficient feature extraction in complex mining environments. The proposed UCM-Net architecture is shown in Figure 1.

The network consists of the following components: the image input, the backbone, the neck, and the head. The backbone extracts features from objects identified by multi-scale detection in underground environments; the neck further integrates these features; finally, the head predicts the class and location of each object.

- Backbone: We set the input image size to 640 × 640 × 3. The HGStem module from HGNetv2 [35] is introduced to capture basic visual features from the input image. Then, the Hierarchical Global Response Normalization Block (HGRNBlock) is used for further feature extraction from underground images. To enhance lightweight processing, we integrate depthwise separable convolution (DWConv) [36] to apply a downsampling operation to feature maps. Finally, the Spatial Pyramid Pooling Fast (SPPF) module is used to concatenate feature maps generated by maximum-pooling operations with different kernel sizes. This integration of multi-scale feature information enhances ESFENet’s ability to extract relevant features effectively.
- Neck: We adopt the FPN + PAN structure for feature fusion, focusing on multi-scale object prediction. The Feature Pyramid Network (FPN) adds a horizontal connection to the backbone, creating an upsampling path that merges low-level and high-level features, enhancing multi-scale detection and semantic information. The Path Aggregation Network (PAN) transfers strong localization information from low-level features and combines it with the semantic information from FPN. It utilizes both horizontal and vertical paths to fuse features of different resolutions, facilitating position information transmission.The PAN implementation includes two C2F modules and two 3 × 3 convolutions, which process features and reduce the feature map size from 80 × 80 to 20 × 20 while maintaining feature connectivity. Within the C2F module, the feature map first undergoes channel adjustment through a 1 × 1 convolution, followed by a split operation along the channel dimension, dividing it into two sub-feature maps. One part retains the original features, while the other uses multiple Bottleneck structures for deep feature extraction. Finally, the two branches are fused through a Concat operation. The C2F modules utilize additional gradient flow branches to enhance the model’s gradient propagation, mitigate gradient vanishing issues, and allow better feature fusion of the multi-scale information extracted by the backbone.
- Head: There are three detection heads in total. Each head is composed of two branches, with each branch containing two CBS layers and a 2D convolution. These branches predict the object’s class and location, respectively.

#### 2.1.2. ESFENet

**Figure 2:**
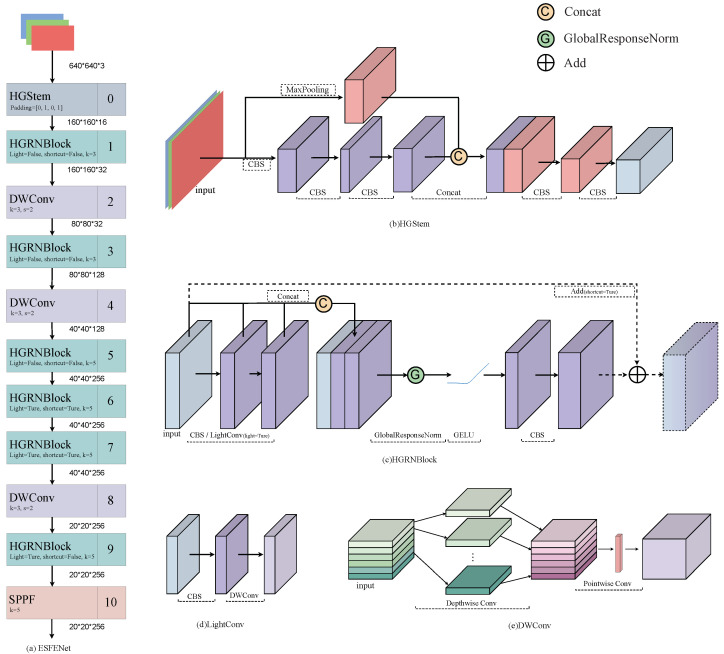
The detailed structure of ESFENet.

As shown in Figure 2a, the structure of the Efficient and Stable Feature Extraction Network (ESFENet) is divided into four stages, progressively increasing the number of channels and the downsampling rates to achieve efficient feature extraction. It includes the initial HGStem module, HGRNBlock modules at each stage, and the DWConv depthwise convolution layers. Some of the HGRNBlock modules utilize LightConv and Add operations to lighten the model and fuse features across stages. Finally, the SPPF module is used in Stage 4 to further process the features. The overall design emphasizes multi-level feature extraction and gradual refinement of the feature maps.

As shown in Figure 2b, HGStem achieves efficient feature extraction and fusion through five convolutional layers and a max-pooling layer. The module performs initial feature extraction with a 3 × 3 convolution and then applies two layers of 2 × 2 convolutions and max-pooling to achieve multi-scale feature fusion. Specifically, the initial feature map is processed by the 3 × 3 convolution layer (stem 1) and then split into two paths: one path goes through the 2 × 2 convolution layers (stem 2a and stem2b) to extract local features, while the other path uses a max-pooling layer to extract global features. The outputs of both paths are then concatenated along the channel dimension. The concatenated result is further processed with a 3 × 3 convolution layer (stem 3) and finally passed through a 1 × 1 convolution layer (stem 4) to generate the output feature map, Fs. The above process can be mathematically represented as follows:

(1) $$F_{s}\left(x\right)=\mathrm{stem}4\left(\mathrm{stem}3\left(\left[\mathrm{stem}2b\left(\mathrm{stem}2a\left(\mathrm{stem}1(x)\right)\right)\right);\mathrm{Maxpool}\left(x\right)\right]\right)$$

The HGStem module is designed to efficiently process low-level features with minimal computational cost. The primary motivation for introducing HGStem in this work is to reduce the model’s computational cost while quickly extracting shallow features from underground mine images. In the HGNetv2 model, the HGBlock module is optimized for GPU computation, making extensive use of 3 × 3 standard convolutions to increase computational efficiency. However, this approach lacks the ability to effectively handle complex mining image features, especially in challenging underground environments. To address this, we redesigned the HGBlock by introducing Global Response Normalization (GRN) and GELU [37] activation functions, creating the HGRNBlock module. This redesign increases the feature extraction capability of the module while maintaining a low computational cost.

As shown in Figure 2c, the HGRNBlock module begins with an input tensor and passes through a series of convolution layers (CBS/LightConv) to better extract various features from the input. These features are then concatenated to aggregate the diverse extracted features. The concatenated feature map is processed with the GRN layer and GELU activation function to enhance the stability of feature extraction. Additionally, squeeze and excitation convolution layers further transform the features to refine feature extraction. The module also includes an optional shortcut connection that allows for residual connections if the input and output channels match. This ensures efficient gradient flow and feature reuse, improving model performance and robustness.

The HGRNBlock effectively combines convolution operations, global normalization, activation functions, and residual connections to form an efficient and stable feature extraction module. Global Response Normalization (GRN) is a technique used for feature normalization in deep learning models. It helps improve the model’s stability and performance, especially in processing image data. Through the introduction of a GRN layer, the model becomes more adaptable to variations in input data, such as different lighting conditions or unseen data, allowing it to perform well even in challenging environments. The main principle of GRN consists of three parts:

1. Global L2 Norm Calculation
  The first step is to compute the global L2 norm for each channel of the input feature map $G_{x}$. This operation calculates the L2 norm for each channel across the entire batch, and the resulting dimensions of *G_x_* will be B × C × 1 × 1, where B is the batch size and C is the number of channels. This step ensures that the norm is calculated independently for each channel while keeping the same dimensions for subsequent operations. The formula for computing G*_x_* is as follows:
  (2) $$G_{x}=\sqrt{\sum_{h=1}^{H}\sum_{w=1}^{W}x_{b,c,h,w}^{2}}$$
2. Normalization Factor Calculation
  Next, the normalization factor *N_x_* is computed by dividing each channel’s norm *G_x_* by the mean of its norms across the channel dimension. This ensures that each channel’s norm is normalized to its own average:
  (3) $$N_{x}=\frac{G_{x}}{\mathrm{mean}{\left(G_{x}\right)}_{C}+\epsilon }$$
  where mean(*G_x_*)*_C_* is the mean of *G_x_* computed across the channel dimensions. ϵ is a small constant added to prevent division by zero. This normalization ensures that each feature channel is scaled according to its global norm, promoting better convergence during training.
3. Scaling and Shifting
  Once the normalization is complete, the feature map is subjected to a scaling and shifting operation. Each normalized feature map is multiplied by a learnable scaling factor γ and added to a learnable shifting factor β, which allows the model to adjust the normalized feature map to better fit the task:
  (4) $$y=\gamma \cdot (x\cdot N_{x})+\beta +x$$
  Here, γ and β are learnable parameters with the same dimensions as the input feature map x. This scaling and shifting operation provides the model with flexibility to adapt the normalized features.
  This residual connection helps ensure the efficient flow of information during training, prevents vanishing gradients, and promotes feature reuse.
  After applying GRN, we introduce the GELU activation function. GELU combines the advantages of the ReLU and sigmoid activation functions. Similar to ReLU, GELU introduces non-linearity, but unlike ReLU, it smoothly handles negative values instead of setting them to zero. This smooth behavior helps the model avoid abrupt changes and better capture complex patterns in the data. The mathematical expression for GELU is as follows:
  (5) GELU(x)=x·Φ(x)
  where Φ(*x*) is the Cumulative Distribution Function (CDF) of the standard normal distribution; it is defined as
  (6) $$\Phi \left(x\right)=\frac{1}{2}\left(1+\mathrm{erf}\left(\frac{x}{\sqrt{2}}\right)\right)$$
  where erf(*x*) is the error function, which is used to calculate the cumulative probability of a normally distributed variable.
  The motivation for adding the GELU activation function after GRN is as follows:
  - Enhancing the Model’s Expressive Power
    GRN (Global Response Normalization) is essentially a normalization operation designed to standardize feature maps, ensuring that the feature values are consistent and enhancing the model’s stability. However, normalization is inherently a linear operation, and such operations may limit the model’s expressive capability. To increase the model’s expressive power, a non-linear activation function such as GELU is introduced after GRN. The introduction of GELU not only increases the model’s ability to represent complex patterns but also ensures smooth handling of features, avoiding the hard cutoff that occurs with functions such as ReLU, which sets negative values to zero. This makes the model more flexible when handling complex data.
  - Preserving Statistical Properties of Feature Maps
    After GRN normalization, the output feature maps exhibit certain statistical properties. To preserve these properties and further enhance the model’s adaptability to different data, it is crucial to select an activation function that aligns with these statistical characteristics. The GELU (Gaussian Error Linear Unit) activation function applies the Cumulative Distribution Function (CDF) of a Gaussian distribution, which is more compatible with the statistical properties of the feature maps processed by GRN. The Gaussian distribution better matches the distribution of the feature maps, helping to maintain their statistical characteristics and improving the model’s performance across a wider range of inputs.

As shown in Figure 2c, in the HGRNBlock module, layers 5, 6, 7, and 9 use the LightConv convolution instead of regular convolutions. As shown in Figure 2d, in LightConv, the number of parameters comes from two parts: 1 × 1 convolution and depthwise separable convolution. Compared to using traditional 3 × 3 or 5 × 5 convolutions directly, LightConv significantly reduces the number of parameters.

In ESFENet, downsampling is also achieved using depthwise separable convolution, which significantly reduces the model’s parameters and computational load. As shown in Figure 2e, the core idea of DWConv (depthwise separable convolution) is to decompose the traditional convolution operation into two independent operations: depthwise convolution and pointwise convolution. In depthwise convolution, each input channel is convolved independently with its own separate filter. In pointwise convolution, a 1 × 1 convolution kernel is used to convolve the output of the depthwise convolution.

By using DWConv, both the parameter count and computational complexity are greatly reduced, making the model more efficient while still capturing important spatial features from the input data.

### 2.2. Self-Supervised Pretraining for Object Detection Tasks

In the field of deep learning for image processing, supervised pre-training based on large-scale labeled datasets (e.g., ImageNet) has become the standard paradigm for model initialization [38]. Pre-training on ImageNet enables the network to learn general visual representations (such as basic textures, shapes, and semantic information) through classification tasks. This type of weight transfer can accelerate the convergence of downstream tasks and improve accuracy. However, it requires a large number of labeled images, and its effectiveness depends on the domain consistency between the source domain (pre-training dataset) and the target domain (downstream task dataset). Given the significant differences between mine datasets and datasets such as ImageNet and COCO, this study employs 110,401 underground coal mine images as the pre-training dataset and performs self-supervised pre-training from scratch.

#### 2.2.1. Hierarchical Masked Encoder and Decoder

**Figure 3:**
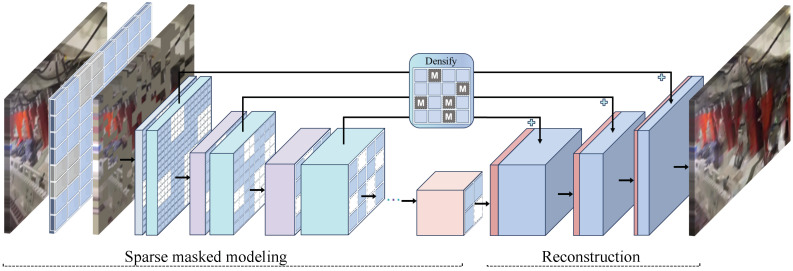
Self-supervised image masking network with ESFENet as an encoder.

To implement self-supervised pre-training on convolutional networks, this paper employs the Sparse Masked Modeling (SparK) method. SparK divides the image into non-overlapping square patches, each of which is independently masked according to a hyperparameter called the “mask ratio”. The unmasked patches are aggregated into a sparse image. As shown in Figure 3, using SparK, we take the proposed ESFENet backbone network as an example and use ESFENet as the self-supervised pre-training encoder. ESFENet is divided into four stages, and for an image of size H × W, ESFENet generates feature maps at four different scales with resolutions of $\frac{H}{4}\times \frac{W}{4}$, $\frac{H}{8}\times \frac{W}{8}$, $\frac{H}{16}\times \frac{W}{16}$, and $\frac{H}{32}\times \frac{W}{32}$, respectively. The entire process uses sparse convolution, where computation is only performed when the center of the sliding window kernel is covered by non-zero elements, skipping all masked regions.

At different stages, the blank elements in the feature map are filled with learnable mask embeddings. The mask embeddings adjust the mask template based on the resolution size and are then processed by a decoder to obtain dense feature maps. This process is referred to as “densification”. The decoder uses a U-Net [39] structure with three upsampling blocks, handling the feature maps from the encoder at four different resolutions, and ultimately reconstructs the image to its original resolution. Between the encoder and decoder at the same resolution, skip connections are present, where layers 1, 3, and 7 of ESFENet and the corresponding U-Net layers have an Add operation. This improves the quality of image reconstruction and further enhances the importance of the encoder in image reconstruction, promoting the encoder’s role in downstream tasks. The model is trained using the L2 loss between the predicted and original images, and the loss is computed only at the masked locations.

#### 2.2.2. Structure Optimization and Downstream Transfer

**Figure 4:**
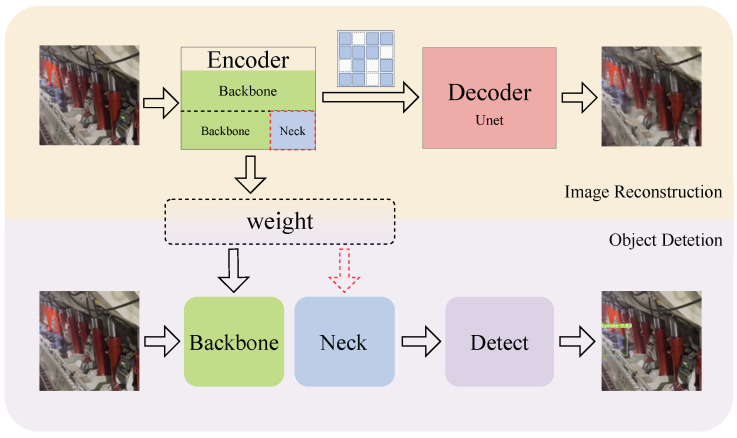
Illustration of the self-supervised pre-training and fine-tuning process.

As shown in Figure 4, after pre-training, the encoder part extracts a large amount of feature information from the underground mining images. The weights of the encoder can be directly used for downstream mining object detection tasks, thereby improving the accuracy of the object detection task. For underground object detection, we propose incorporating the Neck part of the detection network into the self-supervised pre-trained encoder, i.e., using both the Backbone and the Neck as the encoder. The motivation behind this approach is as follows: First, the Neck combines features from multiple scales, which allows it to produce a richer and more informative representation, enhancing the encoder’s ability to capture spatial relationships and contextual information. This is crucial for tasks that require detailed scene understanding. Additionally, from the SparK method paper [32], we conclude that as the encoder’s parameter size decreases, the effectiveness of self-supervised pre-training diminishes. The model used in our underground detection tasks has a smaller parameter size and computation cost than the smallest model shown in the paper. Adding the Neck part to the encoder increases the model parameters in the encoder, which enhances the effectiveness of self-supervised pre-training for detection tasks.

## 3. Experiment Preparation

### 3.1. Datasets

To thoroughly validate the effectiveness and generalization capability of the proposed universal detection network for underground mines, and to ensure that the self-supervised pre-trained model can fully capture the features of mining images, we utilized a dataset from the underground longwall mining face [40] (DsLMF+), which consists of six task-specific datasets: coal mine workers, hydraulic support shields, large coal blocks, safety helmets, miner behaviors, and drag chains, totaling 138,004 images. The dataset’s usability and accuracy have been reviewed and verified by experts in the field of coal mining. Since the safety helmet dataset contains only one class (safety helmets) without negative samples, we chose to use the remaining five datasets. Detailed information is provided in Table 1: 30,704 images of mine workers, sourced from 58 different scenes; 21,017 images of large coal blocks, sourced from 18 different scenes; 21,412 images of drag chains, sourced from 65 different scenes; 24,709 images of miner behaviors, covering eight categories such as sitting, operating devices, leaning against objects, and climbing over obstacles, sourced from 67 different scenes; and 20,045 images of hydraulic support shields, including nine categories covering both normal and abnormal states of coal mining machines and hydraulic support shields from various angles, sourced from 159 different scenes. These five datasets were split into training, validation, and test sets with a ratio of 7:1:2, and the performance of the model was evaluated based on the results from the test set. To ensure the effectiveness of self-supervised pre-training, we used the training and validation sets from these datasets as the pre-training data.

### 3.2. Implementation Details

The hardware configuration used in the experiment includes an Intel Core i7-6700K CPU, an NVIDIA GeForce RTX 3080 GPU, and the operating system is Windows 10. The software configuration consists of CUDA 11.8 and cuDNN 8.5.0. The network model is built using Python 3.10 and PyTorch 2.0.1. The training strategy parameters for the object detection experiments in this paper are shown in Table 2, while the training strategy parameters for self-supervised pre-training are shown in Table 3. To ensure the fairness of the experiments, the experiments involving the design of the mine network structure model do not use pre-trained weights, while the experiments validating the effect of self-supervised pre-training all load the pre-trained weights uniformly.

### 3.3. Evaluation Metrics

To comprehensively evaluate the performance of the object detection model for underground mines, we use commonly employed metrics including Mean Average Precision (mAP) and F1-score. Average Precision (AP) is the area under the precision–recall curve for a specific class. AP integrates precision values across all recall levels, effectively summarizing the model’s performance for that class, while mAP is the mean of the AP values for all classes. The F1-score is another key metric, especially for imbalanced datasets where the frequency of positive class instances is much lower than that of the negative class. The F1-score is the harmonic mean of precision and recall, balancing the two metrics to provide a single performance score. The formulas for these metrics are as follows:

(7) $$AP=\int_{0}^{1}P\left(R\right)dR$$

(8) $$mAP=\frac{1}{N}\sum_{i=1}^{N}AP_{i}$$

(9) $$F1=2\times \frac{P\times R}{P+R}$$

where i represents a specific class, AP(i) denotes the detection precision for that class, mAP is the average of detection precisions across all classes, n is the total number of classes, P represents precision, and R represents recall.

By using the mAP and F1-score metrics, the evaluation of the object detection model becomes more comprehensive. Specifically, mAP ensures robustness by considering multiple IoU thresholds, while F1-score balances the trade-off between precision and recall. To effectively evaluate model complexity and computational resource consumption, we introduce two additional metrics: the number of parameters and the computational cost.

## 4. Experimental Results and Discussion

### 4.1. The Experiment of Detection

#### 4.1.1. Comparison Experiments

A detailed comparative experiment was conducted between classic one-stage and two-stage object detection models, as well as the latest YOLO series models. To provide a unified representation of model performance, we used the COCO metrics, including AP, AP_50_, and AP_75_, for algorithm performance comparison. The experimental results are shown in Table 4. One-stage detection algorithms outperform two-stage algorithms in both precision and computational efficiency. The latest YOLO series algorithms maintain high precision while keeping low parameter count and computational cost for the mine detection task. UCM-Net outperforms all other models in terms of detection metrics on the Support Guard Plate and Miners’ Behaviors datasets. On the Coal Miners dataset, UCM-Net’s AP_50_ and AP_75_ are slightly lower than those of YOLOv5s, with AP_50_ being 0.2% lower and AP_75_ 0.5% lower. However, YOLOv5s has 3 times the parameter count and more than twice the computational cost of UCM-Net. On the Large Coal dataset, UCM-Net achieves better AP and AP_50_ than all other models, although its AP_50_ is 0.6% lower than that of YOLOv5s and YOLOX-s. On the Drag Chain dataset, UCM-Net also outperforms all other models in terms of AP and AP_50_, with its AP_50_ being comparable to other models.

As shown in Table 4, the YOLO algorithms perform exceptionally well on mine detection tasks, so all subsequent experiments were conducted using YOLO models. For easier comparison of results, we standardized the calculation of mAP_50:95_, mAP_50_, F1-score, Precision and Recall using the YOLO metrics.

**Figure 5:**
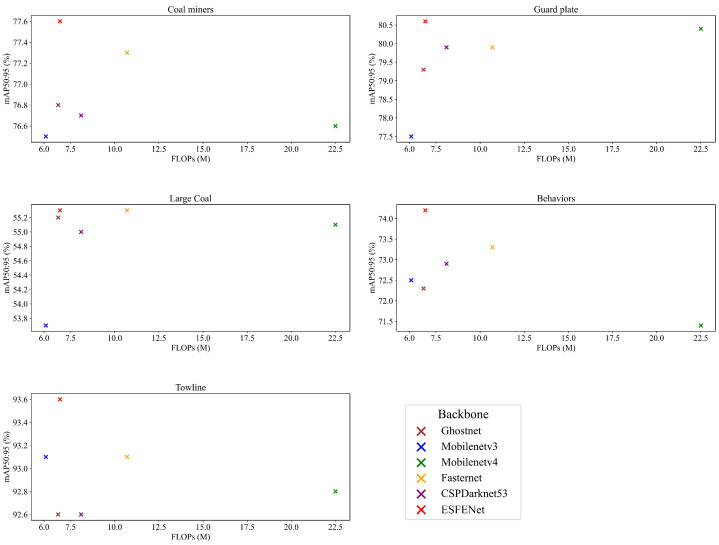
FLOPs vs. mAP: Performance Comparison of Lightweight Backbones on Five Mine Datasets.

To verify that the proposed ESFENet network structure combines lightweight design with strong feature extraction capabilities for mine detection tasks, we compared it with various classic and the latest lightweight backbones, as shown in Table 5 and Figure 5. Although on the Coal Miners, Support Guard Plate, and Drag Chain datasets, ESFENet’s F1-score is slightly lower than that of Fasternet [41], CSPDarknet53, and Ghostnet [42], and ESFENet’s computational cost is slightly higher than Mobilenetv3 [43], overall, ESFENet achieves the best performance. It successfully combines lightweight design while enhancing accuracy.

As demonstrated in Table 6, we expanded the comparative experiments between UCM-Net and the latest YOLO series models in terms of precision, recall, and inference speed (tested on an NVIDIA 3080 GPU). On the “Coal miners” dataset, UCM-Net achieves a precision of 96.3%, surpassing all competing models, while its recall of 95.3% matches YOLOv8’s 95.5%, attesting to its robust performance. For the “Guard plate” dataset, UCM-Net attains 95.6% precision, outperforming other models, with a recall of 92.5% slightly below YOLOv10’s 93.7%, yet maintaining balanced overall performance. Notably, on the “Large Coal” dataset, UCM-Net achieves a 74.9% recall rate, significantly exceeding all baseline models (YOLOv8: 74.1%, YOLOv10: 72.6%, YOLOv12: 73.6%), highlighting its exceptional capability in reducing missed detections. For behavioral recognition tasks (“Behaviors” dataset), UCM-Net delivers 87.0% precision and 88.0% recall, surpassing both YOLOv10 and YOLOv12 while exceeding YOLOv8’s recall of 85.7%. On the “Drag Chain” dataset, UCM-Net maintains 99.6% precision and recall, comparable to other high-performing models with consistent stability. Crucially, UCM-Net achieves 77.1 FPS on an NVIDIA 3080 GPU, substantially outperforming YOLOv10 (70.1 FPS) and YOLOv12 (49.6 FPS), and despite trailing YOLOv8 (96.5 FPS), it effectively balances detection accuracy with real-time efficiency. Collectively, these results underscore UCM-Net’s superior equilibrium between precision and recall, coupled with competitive inference speed, making it particularly suited for coal mine underground environments where simultaneous demands for high-precision detection and real-time responsiveness are critical in industrial inspection applications.

To further validate the capability of our proposed UCM-Net for mine detection tasks, we compared it with the baseline models and YOLOv10 model. As shown in Table 7, UCM-Net demonstrates a lower false detection rate and more accurate bounding box predictions on the Coal Miners dataset compared to YOLOv8 and YOLOv10. On the Guard Plates and Miners’ Behaviors datasets, UCM-Net shows better performance in recognizing small objects and lower false detection rates. On the Large Coal dataset, UCM-Net has lower false detection and missed detection rates. On the Drag Chains dataset, the results are similar across models. The example results in the figure correspond to the data presented in Table 4.

#### 4.1.2. Ablation Experiments

To validate the effectiveness of each module in the proposed ESFENet and explore their interaction effects, we conducted an ablation study, as shown in Table 8. The results demonstrate that replacing standard convolution with depthwise separable convolution (DWConv) effectively reduces the number of parameters and computational complexity while maintaining or even slightly improving detection accuracy, indicating that DWConv eliminates redundant computations while preserving essential feature extraction capabilities. Similarly, replacing C2f with HGRNBlock significantly enhances feature representation and normalization stability while ensuring model lightweight characteristics, benefiting from multi-branch convolution, GRN normalization, and GELU smooth activation. Furthermore, the combination of DWConv and HGRNBlock integrates efficient lightweight feature extraction with strong normalization capabilities, achieving a superior balance between performance and efficiency, which fully demonstrates the synergistic benefits of the proposed improvements. Additionally, the comparison between HGRNBlock and HGBlock reveals that replacing HGBlock with HGRNBlock maintains the same parameter and computational complexity while enhancing the Backbone’s feature extraction capability, ultimately leading to improved accuracy.

#### 4.1.3. Generalization Experiments

To demonstrate the effectiveness of our proposed method for mine detection tasks on other ground-level scene datasets, we used the Pascal VOC 2007 + 2012 dataset, as shown in Table 9. Replacing standard convolutions with depthwise separable convolutions reduces the model’s accuracy, while the HGBlock and HGRNBlock modules improve accuracy while further reducing computational cost. However, the performance of our proposed HGRNBlock module on the Pascal VOC dataset is not as strong as on the mine datasets, indicating that the HGRNBlock module effectively addresses certain challenges in mine image detection. Therefore, our UCM-Net is better suited for mine scene tasks than for other scene types.

### 4.2. Self-Supervised Pre-Training Results on Downstream Tasks

#### 4.2.1. Comparative Experiment

To validate the effectiveness of self-supervised pre-training on mine images, we first conducted experiments comparing the performance of official supervised pre-training weights with our self-supervised pre-training weights on detection tasks. As shown in Table 10, in YOLOv8s, the self-supervised pre-training on the Backbone performs slightly worse than the official supervised pre-training(based on the COCO dataset) weights on the entire YOLOv8s structure for the Coal Miners dataset, but is comparable or even better on other datasets. In YOLOv8n-Fasternet, where we used Fasternet as the Backbone, we loaded both our self-supervised pre-training weights and the official supervised pre-training weights(based on the ImageNet dataset). Table 10 shows that loading only the official pre-trained weights for the Fasternet part actually reduces detection accuracy, while the self-supervised pre-training weights improve detection accuracy. This indicates that due to the significant differences between natural and mine environments, pre-trained weights from natural scene images are not suitable for mine scene tasks. However, our self-supervised pre-training method, based on mine images, can enhance detection accuracy for mine tasks.

To further investigate the effect of self-supervised pre-training on the mine datasets, we conducted experiments focusing on the model parameter size and the self-supervised pre-training mask rate, as shown in Table 10. As the parameter size of the Encoder part in the self-supervised pre-training decreases, the benefit of self-supervised pre-training on detection tasks weakens. In YOLOv8n, self-supervised pre-training based on the Backbone can no longer improve detection accuracy as much as supervised pre-training weights for the entire architecture. Regarding the impact of the masking ratio on self-supervised pre-training, experiments were conducted using two different masking ratios: the optimal 75% masking ratio derived from the experiments by He et al. [29] based on the MAE, and the 60% masking ratio selected in SparK [32]. We found that a 60% masking ratio achieved better results in most experiments, as shown in Table 10.

To address the issue where small models benefit less from self-supervised pre-training, we designed a method to include the Neck structure in the self-supervised pre-training process. As shown in Table 10, when we used Backbone+Neck from YOLOv8n as the Encoder for self-supervised pre-training, detection accuracy improved further compared to pre-training based on Backbone alone. The performance on the Support Guard Plate and Large Coal datasets was even better than with supervised pre-training.

#### 4.2.2. Self-Supervised Pre-Training Experiment on UCM-Net

Based on the above analysis, to further improve the accuracy of the proposed UCM-Net network structure in mine detection tasks and reduce the reliance on supervised data annotation, we conducted self-supervised pre-training on the UCM-Net model using multiple self-supervised pre-training methods. First, we utilized the Backbone+Neck part of UCM-Net as the Encoder for self-supervised pre-training and adopted the sparse hierarchical masking reconstruction method of SparK (with a masking ratio of 60%) to pre-train the model on mine data, achieving promising results. As shown in Table 11, the accuracy was improved on the Coal Miners, Guard Plates, Large Coal, and Miners’ Behaviors datasets. However, for the Drag Chains dataset, as its detection accuracy was already near saturation, the pre-training did not yield significant improvements.Subsequently, we conducted comparative experiments using other self-supervised pre-training methods SimSiam [44] and MoCov2 [45]. The experimental results indicate that SimSiam and MoCov2 failed to effectively enhance detection performance, and even SimSiam exhibited severe “negative transfer” in the downstream detection task. This suggests that SimSiam and MoCov2 did not sufficiently capture key regions or local information during pre-training, as they primarily focused on global invariant features, disregarding the sensitivity to local details crucial for object detection tasks. Consequently, the features obtained through pre-training were not well aligned with the requirements of downstream detection tasks, leading to adverse effects on detection performance.In contrast, the SparK pre-training method, which employs a sparse and hierarchical masking modeling approach, emphasizes local region information reconstruction, enabling the model to more effectively restore local details and multi-scale features. This enhances the detector’s sensitivity and robustness in object localization and recognition tasks. This method not only improves performance in certain mine detection tasks but also provides an effective approach to reducing the effort required for large-scale supervised data annotation.

## 5. Conclusions

In this paper, we conducted research to enhance underground object detection in coal mines from two aspects: model network structure and model pre-training/fine-tuning.

1. The proposed ESFENet algorithm enhances the network’s feature extraction capability to adapt to the complex and dynamic coal mine environment. Ablation experiments show that the average mAP_50:95_ increased by 0.84% across five underground coal mine datasets, and the algorithm’s generalization ability was validated on the Pascal VOC2007 + 2012 dataset.
2. The proposed universal underground coal mine detection model, UCM-Net, was tested on five mining detection datasets. The experimental results demonstrate that UCM-Net improves detection accuracy while reducing parameter size and computation cost. It achieved state-of-the-art (SOTA) performance on all five datasets, with a 21.5% reduction in parameter size and a 14.8% reduction in computational cost. Particularly on the miner behavior dataset, it achieved a 1.3% increase in mAP_50:95_.
3. To address the mismatch between the officially provided supervised pre-training weights and the underground detection tasks, we utilized a self-supervised pre-training method to train a model-specific pre-training weight for underground coal mine detection, further improving the detection model’s accuracy. Experimental analysis shows that when the image masking ratio is 60%, the accuracy improvement on downstream detection tasks is the most significant.
4. As the parameter size of the self-supervised pre-training model decreases, its improvement in detection accuracy becomes lower than that of supervised pre-training methods. To solve this issue, we included both the Backbone and Neck of the model into the pre-training structure as the Encoder, which strengthened the adaptability of self-supervised pre-training to downstream tasks. This resulted in UCM-Net achieving an average mAP_50:95_ of 94.4% across five datasets.

In the future, we will further explore how small models can better benefit from self-supervised pre-training and investigate the construction of a multi-modal large model for underground coal mines. This model will be designed to work in conjunction with lightweight detection models on various edge devices, enabling precise handling of diverse underground conditions.

## Figures and Tables

**Table 1:** Information on datasets.

Dataset	Classes	Total Images	Scenes	Images for Training	Images for Validation	Images for Testing
Coal miners	1	30,704	58	21,492	3071	6141
Support guard plates	9	20,045	159	14,031	2005	4009
Large coal	1	21,017	18	14,711	2102	4204
Miners’ behaviors	8	24,709	67	17,296	2471	4942
Drag chains	1	21,412	65	14,987	2142	4283

**Table 2:** Object detection training strategy parameters.

Configuration Parameter	Value
Optimizer	SGD
Initial learning rate	$1\times 10^{-2}$
Final learning rate	$1\times 10^{-4}$
Momentum	0.937
Weight decay	$5\times 10^{-4}$
Batch size	8
Epoch	400
Input size	640
Close mosaic	10
patience	50

**Table 3:** Self-supervised pre-training strategy parameters.

Configuration Parameter	Value
Input size	640
Batch size	8
Base_lr	$2\times 10^{-4}$
Epoch	400
Sbn	True
Clip	5

**Table 4:** Comparison of COCO metrics for different detection models on the five mine datasets.

Methods	Backbone	AP (%)/AP_50_ (%)/AP_75_ (%)					Params (M)	FLOPs
		Coal Miners	Guard Plates	Large Coal	Behaviors	Drag Chains		
Faster R-CNN	ResNet50	60.7/94.0/65.0	72.8/96.0/85.1	43.3/75.9/43.5	59.3/86.4/69.8	83.1/98.8/92.2	41.35	52.0
YOLOv3	Darknet53	64.5/95.3/74.2	70.6/96.7/80.9	47.5/80.9/51.1	61.6/88.1/74.9	81.0/98.9/93.7	61.52	46.5
YOLOv5-s	CSPDarknet53	74.3/98.2/84.7	79.2/97.3/90.7	52.4/84.1/57.3	71.0/90.6/84.4	90.1/99.0/97.7	7.01	15.8
YOLOX-tiny	CSPDarknet53	73.4/97.5/82.8	78.6/97.2/89.4	53.2/83.4/59.0	69.5/89.6/83.2	88.7/99.0/96.8	5.03	7.6
YOLOX-s	CSPDarknet53	73.0/97.4/82.9	79.2/97.1/90.0	54.2/84.1/60.2	71.2/90.3/84.9	89.5/99.0/96.8	8.94	13.3
YOLOv8n	CSPDarknet53	74.6/98.0/84.1	78.6/97.4/88.6	54.5/83.0/61.5	71.5/90.1/83.9	90.4/99.0/97.7	3.01	8.1
YOLOv10n	CSPDarkent53	74.5/97.7/84.7	78.7/96.9/87.5	54.1/81.9/61.6	70.9/89.7/84.6	90.3/98.9/97.7	2.69	8.2
UCM-Net	ESFENet	74.6/98.0/84.2	79.2/97.4/92.0	54.9/83.5/61.7	72.2/90.6/85.0	91.0 /98.9/97.8	2.35	6.9

**Table 5:** Comparison of different lightweight backbones on the five mine datasets.

Methods	Backbone	mAP_50:95_ (%)/mAP_50_ (%)/F1-Score (%)					Params (M)	FLOPs
		Coal Miners	Guard Plates	Large Coal	Behaviors	Drag Chains		
YOLOv8n	Ghostnet	76.8/98.4/95.6	79.3/97.4/93.1	55.2/84.0/76.9	72.3/89.9/85.8	92.6/99.5/99.7	2.77	6.8
	Mobilenetv3	76.5/98.2/95.5	77.5/97.1/91.9	53.7/83.0/75.8	72.5/90.5/86.4	93.1/99.5/99.6	3.11	6.1
	Mobilenetv4	76.6/98.4/95.6	80.4/97.8/94.1	55.1/83.7/76.6	71.4/90.8/86.0	92.8/99.4/99.4	5.7	22.5
	Fasternet	77.4/98.3/95.9	79.6/97.4/92.8	55.1/83.8/76.8	73.3/90.6/86.7	93.1/99.5/99.6	4.17	10.7
	CSPDarkent53	76.7/98.3/95.7	79.9/97.7/94.3	55.0/83.5/76.3	72.9/90.7/87.3	92.6/99.5/99.5	3.01	8.1
	ESFENet	77.6/98.5/95.8	80.6/97.8/94.0	55.3/84.0/77.0	74.2/91.5/87.5	93.6/99.5/99.6	2.35	6.9

**Table 6:** Performance comparison of UCM-Net and the latest YOLO series models.

Method	Precision (%)/Recall (%)					FPS (Frames/s)
	Coal Miners	Guard Plates	Large Coal	Miners’ Behaviors	Drag Chains	
YOLOv8n	96.0/95.5	95.5/93.1	78.7/74.1	88.9/85.7	99.4/99.6	96.5
YOLOv10n	95.8/94.2	93.3/93.7	78.2/72.6	85.7/85.1	99.2/99.4	70.1
YOLOv12n	96.0/95.5	94.9/92.6	79.4/73.6	85.0/87.2	99.5/99.4	49.6
UCM-Net(OUR)	96.3/95.3	95.6/92.5	79.2/74.9	87.0/88.0	99.6/99.6	77.1

**Table 7:** Visualization results of the UCM-Net, YOLOv8, and YOLOv10 algorithms on different datasets.

	Ground Truth	YOLOv8n	YOLOv10n	UCM-Net (OUR)
Coal Miners	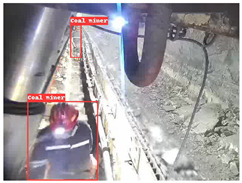	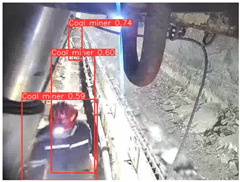	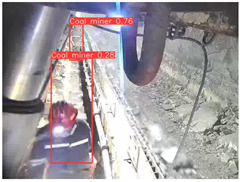	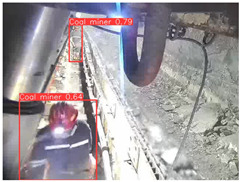
	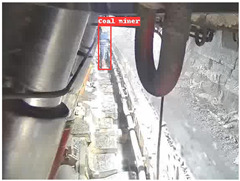	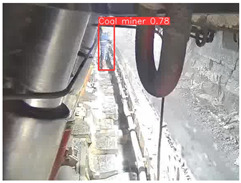	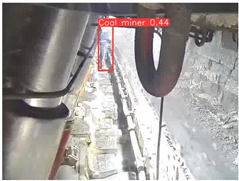	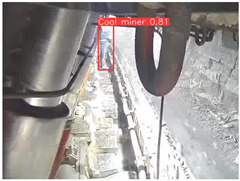
Guard Plates	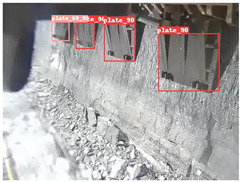	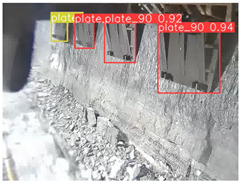	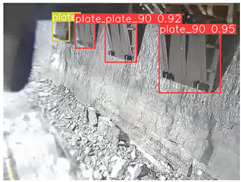	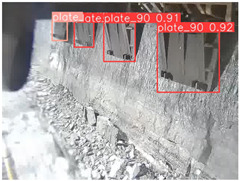
	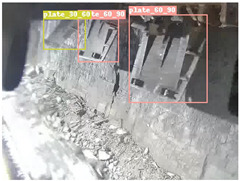	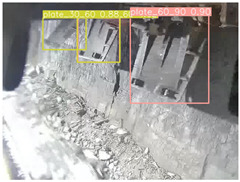	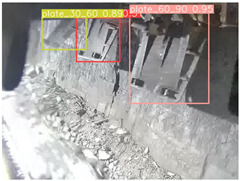	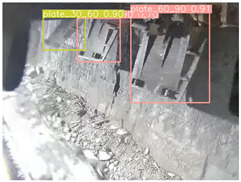
Large Coal	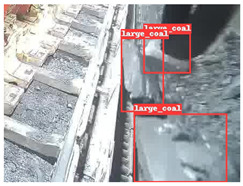	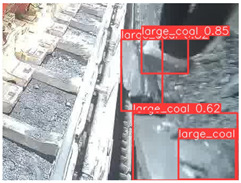	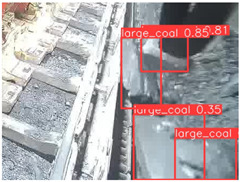	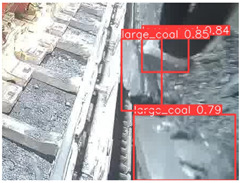
	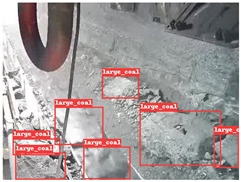	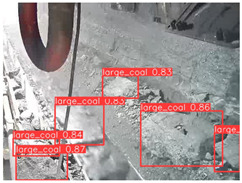	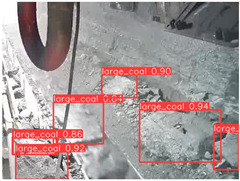	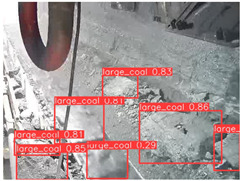
Miners’ Behaviors	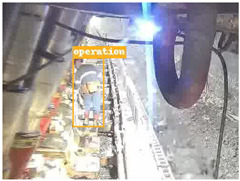	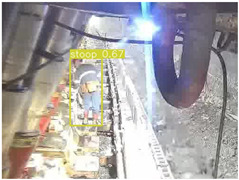	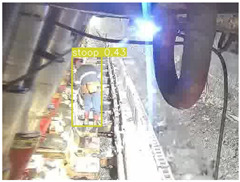	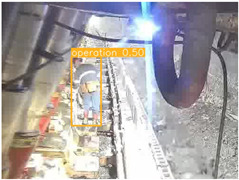
	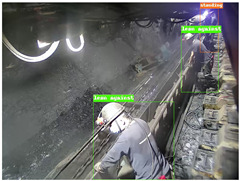	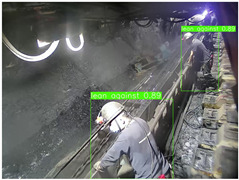	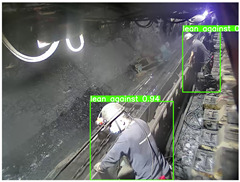	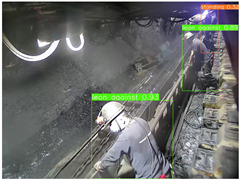
Drag Chains	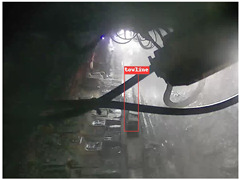	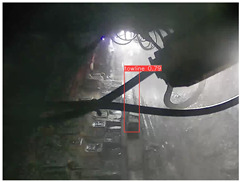	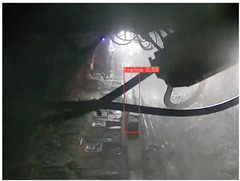	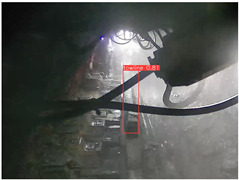
	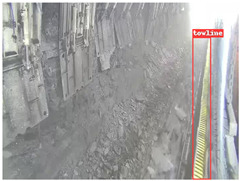	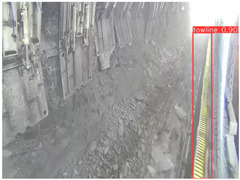	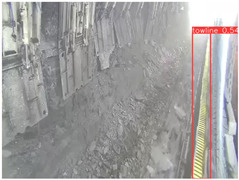	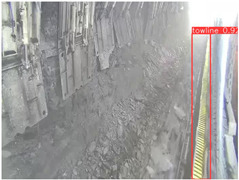

**Table 8:** Ablation experiment on the five mine datasets.

Methods	mAP_50:95_ (%)/mAP_50_ (%)					Params (M)	FLOPs
	Coal Miners	Guard Plates	Large Coal	Miners’ Behaviors	Drag Chains		
Conv+C2f (baseline)	76.6/98.3	79.9/97.7	55.0/83.5	72.9/90.7	92.6/99.5	3.01	8.1
DWConv + C2f	76.7/98.3	80.1/97.8	55.1/83.9	73.8/90.5	92.8/99.4	2.62	7.2
DWConv + HGBlock	77.1/98.4	80.4/97.8	55.2/83.9	73.0/91.1	93.6/99.4	2.35	6.9
DWConv + HGRNBlock × 1	77.2/98.5	80.6/97.9	55.3/84.0	73.9/91.3	93.6/99.4	2.35	6.9
Conv + HGRNBlock × 6	77.5/98.5	81.1/97.8	55.3/83.6	74.5/91.6	93.5/99.4	3.1	8.1
DWConv + HGRNBlock × 6	77.6/98.5	80.6/97.8	55.3/84.0	74.2/91.5	93.6/99.5	2.35	6.9

**Table 9:** Performance of each module on the Pascal VOC 2007+2012 dataset.

Method	mAP_50_	Precision	Recall	F1-Score
Conv + C2f (baseline)	69.8	75.4	62.5	68.3
DWConv + C2f	67.7	74.6	60.3	66.7
DWConv + HGBlock	69.6	75.6	62.2	68.2
DWConv + HGRNBlock × 1	69.7	75.5	62.3	68.3
DWConv + HGRNBlock × 6	69.5	76.8	61.5	68.3

**Table 10:** Effects of different pre-training methods on downstream task models.

Methods	Pre-Training Methods	Migration Component (Params)	Masking Ratio	mAP_50:95_ (%)/mAP_50_ (%)				
				Coal Miners	Guard Plates	Large Coal	Miners’ Behaviors	Drag Chains
YOLOv8s	Random Initialization			78.9/98.7	81.6/97.9	56.2/84.3	74.9/91.4	93.5/99.4
	Official Supervised	Whole Framework (11.1 M)		79.9/98.7	81.8/97.7	56.8/84.7	75.1/91.4	93.9/99.4
	Self-supervised	Backbone (4.4 M)	75%	79.1/98.7	81.7/97.9	56.7/84.7	75.3/91.5	93.7/99.5
		Backbone (4.4 M)	60%	79.2/98.7	82.3/97.7	56.7/84.8	75.7/91.5	93.7/99.5
YOLOv8n- fasternet	Random Initialization			77.4/98.3	79.6/97.4	55.1/83.8	73.3/90.6	93.1/99.5
	Official Supervised	Backbone (2.2 M)		73.6/97.5	79.3/97.6	54.8/83.9	70.4/89.8	91.4/99.5
	Self-supervised	Backbone (2.2 M)	75%	77.6/98.4	81.5/97.7	55.1/83.6	73.7/91.4	93.2/99.4
		Backbone (2.2 M)	60%	77.7/98.4	81.3/97.8	55.4/84.0	73.5/91.1	93.5/99.4
YOLOv8n	Random Initialization			76.6/98.3	79.9/97.7	55.0/83.5	72.9/90.7	92.6/99.5
	Official Supervised	Whole Framework (3.0 M)		78.1/98.6	79.8/97.4	55.8/84.2	74.7/91.2	93.6/99.4
	Self-supervised	Backbone (1.1 M)	75%	77.0/98.5	80.9/97.7	55.7/84.1	73.1/90.7	92.6/99.5
		Backbone (1.1 M)	60%	77.4/98.6	81.1/97.6	55.6/84.0	72.9/91.0	93.0/99.5
		Backbone + Neck (2.3 M)	75%	77.4/98.5	80.9/97.8	55.6/84.0	73.3/91.3	93.0/99.5
		Backbone + Neck (2.3 M)	60%	77.5/98.6	81.3/97.9	55.8/84.2	73.5/90.8	93.1/99.5

**Table 11:** Self-supervised pre-training performance on UCM-Net with a 60% image masking ratio.

Methods	Pre-Training Methods	mAP_50:95_ (%)/mAP_50_ (%)				
		Coal Miners	Guard Plates	Large Coal	Miners’ Behaviors	Drag Chains
UCM-Net	Random Initialization	77.6/98.5	80.6/97.8	55.3/84.0	74.2/91.5	93.6/99.5
	SimSiam	75.8/97.9	76.9/96.4	54.9/83.2	69.3/88.6	90.9/99.4
	MoCov2	77.3/98.5	81.1/97.8	54.9/83.7	73.5/91.6	93.6/99.5
	SparK	77.7/98.6	81.3/97.9	55.8/84.2	74.5/91.9	93.6/99.4

## Data Availability

Data are contained within the article.

## References

[1] Liu H.D., Zhang H., Wang J.P., Dou J.X., Guo R., Li G.Y., Liang Y.H., Yu J.L. (2024). Construction of macromolecular model of coal based on deep learning algorithm. Energy.

[2] Zhang K., Yang X., Xu L., Thé J., Tan Z., Yu H. (2024). Enhancing coal-gangue object detection using GAN-based data augmentation strategy with dual attention mechanism. Energy.

[3] Zhang K., Wang T., Yang X., Xu L., Thé J., Tan Z., Yu H. (2024). STATNet: One-stage coal-gangue detector based on deep learning algorithm for real industrial application. Energy AI.

[4] Wu B., Wang J., Qu B., Qi P., Meng Y. (2023). Development, effectiveness, and deficiency of China’s coal mine safety supervision system. Resour. Policy.

[5] Huang K., Li S., Cai F., Zhou R. (2023). Detection of large foreign objects on coal mine belt conveyor based on Improved. Processes.

[6] Wu X., Li H., Wang B., Zhu M. (2022). Review on improvements to the safety level of coal mines by applying intelligent coal mining. Sustainability.

[7] He D., Le B.T., Xiao D., Mao Y., Shan F., Ha T.T.L. (2019). Coal mine area monitoring method by machine learning and multispectral remote sensing images. Infrared Phys. Technol..

[8] Dou D., Wu W., Yang J., Zhang Y. (2019). Classification of coal and gangue under multiple surface conditions via machine vision and relief-SVM. Powder Technol..

[9] He K., Zhang X., Ren S., Sun J. (2015). Spatial pyramid pooling in deep convolutional networks for visual recognition. IEEE Trans. Pattern Anal. Mach. Intell..

[10] Ren S., He K., Girshick R., Sun J. (2017). Faster R-CNN: Towards real-Time object detection with region proposal networks. IEEE Trans. Pattern Anal. Mach. Intell..

[11] Redmon J., Divvala S., Girshick R., Farhadi A. You only look once: Unified, real-time object detection. Proceedings of the IEEE Conference on Computer Vision and Pattern Recognition.

[12] Redmon J., Farhadi A. (2018). Yolov3: An incremental improvement. arXiv.

[13] Bochkovskiy A., Wang C.Y., Liao H.Y.M. (2020). Yolov4: Optimal speed and accuracy of object detection. arXiv.

[14] Li C., Li L., Jiang H., Weng K., Geng Y., Li L., Ke Z., Li Q., Cheng M., Nie W. (2022). YOLOv6: A single-stage object detection framework for industrial applications. arXiv.

[15] Wang C.Y., Bochkovskiy A., Liao H.Y.M. YOLOv7: Trainable bag-of-freebies sets new state-of-the-art for real-time object detectors. Proceedings of the IEEE/CVF Conference on Computer Vision and Pattern Recognition.

[16] Imam M., Baïna K., Tabii Y., Ressami E.M., Adlaoui Y., Benzakour I., Abdelwahed E.H. (2023). The future of mine safety: A comprehensive review of anti-collision systems based on computer vision in underground mines. Sensors.

[17] Azhari F., Sennersten C.C., Lindley C.A., Sellers E. (2023). Deep learning implementations in mining applications: A compact critical review. Artif. Intell. Rev..

[18] Liu Y., Wang X., Zhang Z., Deng F. (2023). LOSN: Lightweight ore sorting networks for edge device environment. Eng. Appl. Artif. Intell..

[19] Zhang J., Feng Y., Li X., Lang D., Xu Y., Li H.A., Li X. (2024). Safety helmet wearing detection algorithm based on DSM-YOLOx. Res. Sq..

[20] Wang Y., Guo W., Zhao S., Xue B., Zhang W., Xing Z. (2022). A big coal block alarm detection method for scraper conveyor based on YOLO-BS. Sensors.

[21] Rao T., Xu H., Pan T. Pedestrian detection model in underground coal mine based on active and semi-supervised learning. Proceedings of the 2023 8th International Conference on Signal and Image Processing (ICSIP).

[22] Wang Z., Liu Y., Duan S., Pan H. (2023). An efficient detection of non-standard miner behavior using improved YOLOv8. Comput. Electr. Eng..

[23] Wen X., Li B., Wang X., Li J., Wei D., Gao J., Zhang J. (2023). A Swin transformer-functionalized lightweight YOLOv5s for real-time coal–gangue detection. J. Real-Time Image Process..

[24] Xue G., Li S., Hou P., Gao S., Tan R. (2023). Research on lightweight Yolo coal gangue detection algorithm based on resnet18 backbone feature network. Internet Things.

[25] Wang B., Cui H., Yu X., Su Z., Zheng Y. (2025). Research on gangue detection method based on GD-YOLO. Eng. Lett..

[26] Zong G., Yue Y., Shan W. (2024). Optimization study of coal gangue detection in intelligent coal selection systems based on the improved YOLOv8n model. Electronics.

[27] Radford A., Narasimhan K., Salimans T., Sutskever I. (2018). Improving language understanding by generative pre-training. Comput. Sci. Linguist..

[28] Lee J., Toutanova K. (2018). Pre-training of deep bidirectional transformers for language understanding. arXiv.

[29] He K., Chen X., Xie S., Li Y., Dollár P., Girshick R. Masked autoencoders are scalable vision learners. Proceedings of the IEEE/CVF Conference on Computer Vision and Pattern Recognition.

[30] Dai Z., Cai B., Lin Y., Chen J. Up-detr: Unsupervised pre-training for object detection with transformers. Proceedings of the IEEE/CVF Conference on Computer Vision and Pattern Recognition.

[31] Carion N., Massa F., Synnaeve G., Usunier N., Kirillov A., Zagoruyko S. (2020). End-to-end object detection with transformers. Proceedings of the European Conference on Computer Vision.

[32] Tian K., Jiang Y., Diao Q., Lin C., Wang L., Yuan Z. (2023). Designing bert for convolutional networks: Sparse and hierarchical masked modeling. arXiv.

[33] Woo S., Debnath S., Hu R., Chen X., Liu Z., Kweon I.S., Xie S. Convnext v2: Co-designing and scaling convnets with masked autoencoders. Proceedings of the IEEE/CVF Conference on Computer Vision and Pattern Recognition.

[34] Liu Z., Mao H., Wu C.Y., Feichtenhofer C., Darrell T., Xie S. A convnet for the 2020s. Proceedings of the IEEE/CVF Conference on Computer Vision and Pattern Recognition.

[35] Zhao Y., Lv W., Xu S., Wei J., Wang G., Dang Q., Liu Y., Chen J. Detrs beat YOLOs on real-time object detection. Proceedings of the IEEE/CVF Conference on Computer Vision and Pattern Recognition.

[36] Chollet F. Xception: Deep learning with depthwise separable convolutions. Proceedings of the IEEE Conference on Computer Vision and Pattern Recognition.

[37] Hendrycks D., Gimpel K. (2016). Gaussian error linear units (gelus). arXiv.

[38] LeCun Y., Bengio Y., Hinton G. (2015). Deep learning. Nature.

[39] Ronneberger O., Fischer P., Brox T. (2015). U-net: Convolutional networks for biomedical image segmentation. Proceedings of the Medical Image Computing and Computer-Assisted Intervention–MICCAI 2015: 18th International Conference.

[40] Yang W., Zhang X., Ma B., Wang Y., Wu Y., Yan J., Liu Y., Zhang C., Wan J., Wang Y. (2023). An open dataset for intelligent recognition and classification of abnormal condition in longwall mining. Sci. Data.

[41] Chen J., Kao S.H., He H., Zhuo W., Wen S., Lee C.H., Chan S.H.G. Run, don’t walk: Chasing higher FLOPS for faster neural networks. Proceedings of the IEEE/CVF Conference on Computer Vision and Pattern Recognition.

[42] Han K., Wang Y., Tian Q., Guo J., Xu C., Xu C. Ghostnet: More features from cheap operations. Proceedings of the IEEE/CVF Conference on Computer Vision and Pattern Recognition.

[43] Howard A., Sandler M., Chu G., Chen L.C., Chen B., Tan M., Wang W., Zhu Y., Pang R., Vasudevan V. Searching for mobilenetv3. Proceedings of the IEEE/CVF International Conference on Computer Vision.

[44] Chen X., He K. Exploring simple siamese representation learning. Proceedings of the IEEE/CVF Conference on Computer Vision and Pattern Recognition.

[45] Chen X., Fan H., Girshick R., He K. (2020). Improved baselines with momentum contrastive learning. arXiv.

